# A Systematic Review of the Relationship between Social Isolation and Physical Health in Adults

**DOI:** 10.3390/healthcare12111135

**Published:** 2024-06-01

**Authors:** Deborah Witt Sherman, Alliete Rodriguez Alfano, Fernando Alfonso, Carmen R. Duque, Daniella Eiroa, Yamile Marrero, Teresa Muñecas, Erica Radcliffe-Henry, Ana Rodriguez, Chelsea L. Sommer

**Affiliations:** 1 Department of Graduate Nursing, Nicole Wertheim College of Nursing and Health Sciences, Miami, FL 33199, USA; 2 Department of Communication and Science Disorders, Nicole Wertheim College of Nursing and Health Sciences, Miami, FL 33199, USA; aalfano@fiu.edu (A.R.A.); csommer@fiu.edu (C.L.S.); 3 Department of Nurse Anesthesiology, Nicole Wertheim College of Nursing and Health Sciences, Miami, FL 33199, USA; falfonso@fiu.edu; 4 Department of Undergraduate Nursing, Nicole Wertheim College of Nursing and Health Sciences, Miami, FL 33199, USA; caduque@fiu.edu; 5 Department of Athletic Training Services, Nicole Wertheim College of Nursing and Health Sciences, Miami, FL 33199, USA; deiroa@fiu.edu; 6 Department of Health Services Administration, Nicole Wertheim College of Nursing and Health Sciences, Miami, FL 33199, USA; ymarrero@fiu.edu; 7 Department of Clinical Education, Nicole Wertheim College of Nursing and Health Sciences, Miami, FL 33199, USA; tmunecas@fiu.edu; 8 Department of Physical Assistant, Hubert Wertheim College of Medicine, Miami, FL 33199, USA; eradclif@fiu.edu; 9 Department of Occupational Therapy, Nicole Wertheim College of Nursing and Health Sciences, Miami, FL 33199, USA; acaldero@fiu.edu

**Keywords:** social isolation, physical health, adults

## Abstract

**Background:** According to the World Health Organization, social isolation, particularly of older adults, is a public health issue endangering the well-being of individuals, families, and communities. Social isolation affects health through biological, behavioral, and psychological pathways and is associated with physical and psychological/emotional well-being, increases morbidity and mortality rates, and lowers quality of life. **Purpose:** This systematic review examined the relationship between social isolation and physical health, including subjective and objective dimensions, and factors that influence this relationship in adults. **Methods**: This systematic review examined six electronic databases covering the field of health and human services and included results from 1 January 2017 to 10 March 2023 with key terms including adult social connection or social isolation coupled with health, physical, psychological, emotional, mental, or behavioral. The initial search yielded 925 research articles across all databases and was narrowed to 710 when the decision was made to focus on social isolation and physical health. Covidence was used throughout the retrieval and appraisal process, as provided in a PRISMA flow diagram. Twenty-four studies that scored 90 or above in the appraisal process were included in the systematic review. **Results**: The studies represented included seven studies conducted in the United States and seventeen studies conducted internationally. Regarding study design, twenty-three studies were quantitative, one was qualitative, and one was mixed methods. The majority of quantitative studies were correlational in design with nine being longitudinal. The majority of studies were based on large national data sets representing in total 298,653 participants aged 50 and older. The results indicate that social isolation is related to increases in inflammatory biomarkers associated with diseases, all-cause mortality, lower expectations of longevity, and frailty. In addition, social isolation was associated with cognitive decline and disruptions in sleep. Poor oral health increased social isolation. The results further indicated that decreased physical performance/function and a decline in physical activity were associated with social isolation, as well as decreased overall physical health, poor health behaviors, and self-care, and decreased health-related quality of life. Further research is warranted to examine the possible bidirectionality of these relationships and possible mediating, moderating, or confounding variables. **Implications**: Future research is needed to explore the biological and behavioral pathways in which social isolation negatively impacts physical health. Going forward, studies are needed that move beyond descriptive, exploratory methods and integrate data from qualitative and mixed-method designs that will inform the development and testing of a conceptual framework related to social isolation and health. By advancing the science behind social isolation, comprehensive interventions can be identified and tested with implications at the individual, family, community, and societal levels to reduce social isolation, particularly among adults, and improve health and quality of life.

## 1. Introduction

Human beings, inherently social creatures, actively pursue connections and social interactions, leveraging their social networks as invaluable resources. Nonetheless, a troubling statistic emerges, with one in five adult Americans reporting feelings of social isolation despite this innate inclination towards sociability [1]. The World Health Organization [2] states that social isolation of older adults has become a public health issue, endangering the financial and physical well-being of individuals, families, and the community. The societal ramifications of social isolation include the need for greater healthcare, pharmaceuticals, and financial resources [3]. Theeke [4] highlights that a number of factors, including a faster-aging population, smaller families, and altered intergenerational relationships, have contributed to the loss of older individuals’ social functions and an increase in social isolation. Over 40% of geriatric patients suffer social isolation because of shifts in social relationships, including geographic migration, and the death of family members [5]. 

According to Zhang and Liu [6], social isolation can be either an active or passive separation from society that limits social engagement, active participation, and interpersonal communication and can have detrimental effects on an individual’s physical or mental health. 

Social isolation is an increasingly identified health concern and is associated with psychological and emotional well-being and physical well-being, including cardiovascular disease, stroke, dementia, cognitive decline, and premature mortality [7]. Research indicates that social isolation not only increases morbidity and mortality rates, but also increases depression, suicidal ideation, and anxiety and reduces quality of life [3,8]. Furthermore, in older adults, social isolation is associated with physical functioning [9], falls [10,11], frailty [12,13], and caregiver self-reported health [14]. According to Delerue Matos et al. [15], social isolation is detrimental to an older person, not only because of an increase in all-cause mortality but also an increase in the prevalence and exacerbation of chronic diseases and poorer mental and cognitive performance. 

### 1.1. Definitions and Dimensions of Social Isolation

Social isolation encompasses a multifaceted construct, compromising both subjective and objective dimensions. Subjective social isolation reflects the perceived deficiency in the desired number or quality of relationships, while objective social isolation involves the absence of a spouse, children, or siblings or non-participation in social organizations, clubs, or religious groups [16]. Drinkwater et al. [17] propose that perceived social isolation is made up of emotions of loneliness and insufficient social support, whereas objective social isolation is defined as the absence of social networks, decreased network size, and decreased participation in social activities. Although social isolation and loneliness are frequently discussed together, social isolation is a strong risk factor for loneliness; however, loneliness can arise when social isolation is not present [18]. 

Cacioppo et al. [19] assert that objective isolation is a significant predictor of subjective isolation, which is associated with negative physical and mental health outcomes. Furthermore, the relationship between objective social isolation and health is mediated by subjective social isolation. Subjective social isolation, characterized as a disruption in social connection and belonging, may have an influence on older individuals’ health by affecting not only mental health but also other mechanisms that determine physical health [20]. For example, Fiordelli et al. [21] examined the relationship between objective and subjective social isolation in older Italian adults. Based on a sample of 306 participants over the age of 65, objective and subjective social isolation were not related directly to physical health, but subjective isolation was associated with worse mental health and depression. However, high levels of social isolation were related to lower levels of physical health through the mediation of mental health. 

### 1.2. Pathways of Social Isolation

Social isolation is believed to affect health through biological, behavioral, and psychological pathways [22]. The first pathway is the direct psycho-biological processes that stimulate neuro-endocrine dysregulation, disturbances in autonomic function, blood pressure control, inflammatory responses, and chronic allostatic load [7]. In addition, based on an evolutionary mechanism, inflammation, as a biological response, is increased because of fear that social isolation may increase physical threat [23]. According to Kahn et al. [24], stress brought on by social isolation can cause physiological dysfunction, including elevated cortisol secretion, metabolic activation, and sympathetic nervous system activation, which contributes to difficulty sleeping. Luo et al. [7], based on a sample of 15,000 adults ages 18–89, using mediation analyses, reported that individuals who were socially isolated have significantly increased risk of overall mortality, cardiovascular disease, and mortality from other causes. The extent to which social isolation and mortality were mediated by health behaviors varied by cause of death, leading to increased public health concerns about social isolation as an additional risk factor. 

The second pathway related to social isolation is behavioral, reflected by less healthy lifestyles and behaviors that contribute to increased health risks, such as smoking, alcohol consumption, unhealthy diets, reduced physical activity, and a sedentary lifestyle [25]. Objective social isolation exhibits a robust association with physical health, potentially impeding health-promoting behaviors, whereas subjective dimensions are intricately intertwined with mental health and depression [25]. Elovainio et al. [26] found that increased mortality in socially isolated individuals was related to low socioeconomic status, unhealthy behaviors (smoking, lack of physical activity, alcohol intake, and poor diet), and mental health issues. Naito [27] further reported that mortality in socially isolated people was associated with co-morbidities and poor health behaviors. The psychological pathway through which social isolation acts as an emotional or social stressor triggers an inflammatory cascade [28]. In chronic states of social isolation, biological and psychological pathways exacerbate inflammatory processes that threaten long-term health. 

### 1.3. Risk Factors of Social Isolation

There are numerous risk factors associated with social isolation, including physical risk factors, such as older age, chronic illness, comorbidities, disability, limited mobility, physical decline, and history of falls [29,30]. In addition, communication impairments, such as speech difficulties and hearing loss, are risk factors for social isolation [31], as well as psychological health, including depression [8]. Socio-economic factors that place older adults at higher risk for social isolation include living arrangements, such as living alone or living in rural or remote locations far from family, friends, and available support, as well as those of lower education and who are economically disadvantaged, as financial constraints may limit participation in social activities, community events, memberships in social groups, and access to transportation [20]. Placing older adults at further risk of social isolation are life transitions, such as bereavement, relocation, and retirement, which may result in the loss of social roles, routines, and support networks that impact a person’s sense of belonging and connectedness [20]. Furthermore, cultural norms, language barriers, and discrimination contribute to social isolation among minority groups, such as immigrants and individuals from marginalized communities [20,30].

Examples of risk factors related to social isolation were reported in a study by Jang et al. [8] based on a sample of 2609 Asian Americans, aged 18 to 98, including Chinese, Korean, Indian, Vietnamese, Filipino, and other Asians. The study investigated the relationship among sociodemographic variables (age, gender, ethnic origin, marital status, education, and perceived financial status), health-related chronic medical conditions and self-rated health, immigration variables (number of years living in the United States and English proficiency), and three types of social isolation (isolation from family, friends, or both). The sample’s total social isolation rate varied from 18.2 to 19.3 percent across all three forms of isolation. According to regression analysis, people in their middle and older years, with poor English proficiency, or who had lived in the United States for less than half of their lives demonstrated higher social isolation. The risk of social isolation related to family was greatest for those who were not married, while the risk of social isolation from friends was greatest for those with unmet financial needs. 

Given this introduction/background, which provides readers with context regarding the concept of social isolation, the purpose of this systematic review was to examine the relationship between social isolation and physical health, including social isolation’s subjective and objective dimensions and factors that influence this relationship in adults. Using conceptual knowledge of this relationship, a future goal will be to develop interventions to reduce social isolation in adults and improve their physical health.

## 2. Methods

To be included in this systematic review, studies had to meet the predetermined initial inclusion criteria. The inclusion criteria were as follows: (1) inclusion of adult participants aged 24 and older, which was based on the literature suggesting that younger adults, aged 18 to 23, may have different social situations and experiences as they are entering adulthood in comparison with middle-age or older adults; (2) focus on outcomes related to social isolation and the relationships between physical and/or mental health; (3) written in English; (4) available as full-text; and (5) published in scholarly, peer-reviewed journals. Exclusion criteria were as follows: (1) individuals who were under the age of 24; (2) systematic reviews; (3) non-human participants; (4) studies related to COVID-19; (5) studies related to veteran participant samples; and (6) intervention studies to more narrowly align the topic. 

### 2.1. Search Strategy

A systematic search strategy provided the process/formula to identify studies for inclusion in this review. Computer literature searches were conducted in six electronic databases covering the field of health and human services (i.e., Education Resources Information Center (ERIC), Cumulative Index to Nursing and Allied Health Literature (CINAHL), MEDLINE, PsycINFO, Embase, and Web of Science). Searches were conducted in March of 2023 and included all results from 1 January 2017 to 10 March 2023. Prior to this review, key terms were determined by the research team through the use of synonyms and by exploring the subjects of relevant articles for additional applicable key terms. Included articles contained the term ‘adult*’ in the abstract and terms ‘social connect*’ or ‘social relation’ or ‘isolation,’ coupled with ‘health’ or ‘physical’ or ‘psychological’ or ‘emotional’ or ‘mental’ or ‘behavioral’ in the title. 

### 2.2. Screening

**Figure 1:**
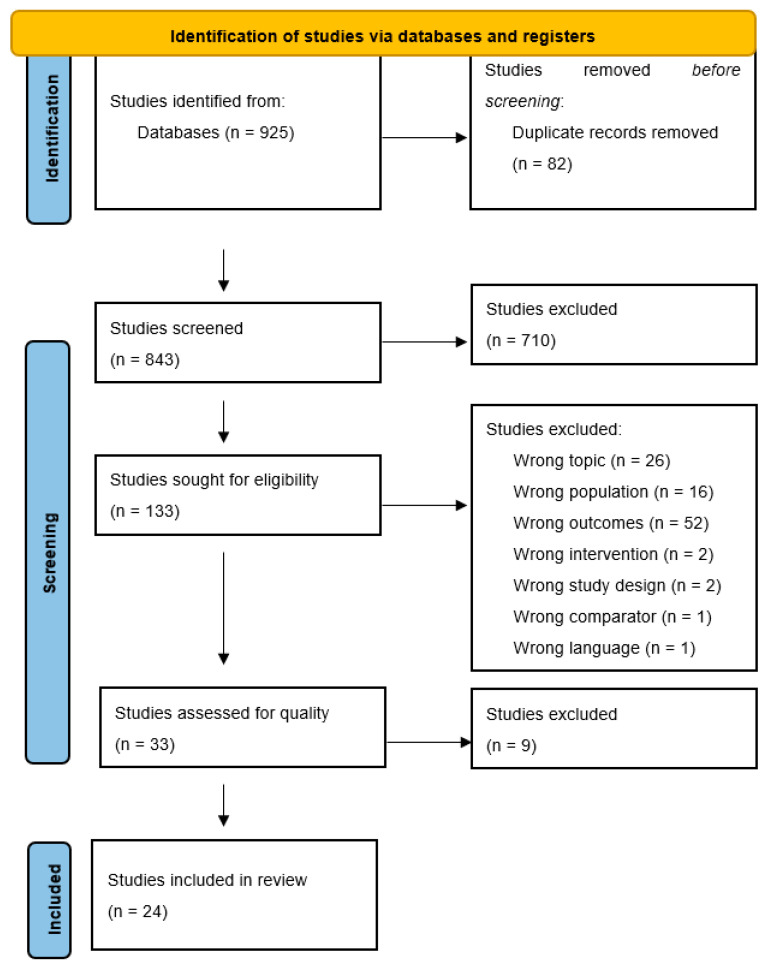
PRISMA flow diagram.

The initial search yielded a combined result of 925 research articles across all identified databases, as seen in the PRISMA flow diagram in Figure 1. To organize and assist with the obtained results, the researchers employed the use of Covidence. This online research screening tool allows researchers to execute the screening process for systematic reviews. Eighty-two duplicates were identified through Covidence upon import and were removed from the total, resulting in 843 research articles to proceed to the screening stage. Each stage of the screening process described below involved two researchers independently and blindly screening each article based on the inclusion and exclusion criteria. In the event of a disagreement, a third researcher screened the article to resolve the disagreement by providing a tie-break vote.

Specifically, the first step of the review process was the screening of the articles by title and abstract relevance, yielding a total of 710 that were excluded as irrelevant. If the title or abstract failed to provide sufficient information to determine eligibility, the article moved to the next stage, the full-text review. One hundred thirty-three articles moved to the full-text review stage and were analyzed to confirm the inclusion criteria. It was at this point that the research team decided to limit the scope of this review by excluding studies related to COVID-19, veterans, and mental/psychological/emotional health, where an additional 100 articles were excluded. Consequently, 33 articles met the established eligibility criteria to be further analyzed for inclusion in this systematic review. 

### 2.3. Study Quality and Potential Sources of Study Bias

Upon completion of the screening process, the remaining 33 articles were critiqued on their strength in relation to the purpose of this study using the procedures outlined by Polit and Beck [32]. Two team members individually examined each study and, following the guidelines of Polit and Beck [32], were given a rating from 0 to 1 (0 = no, 1 = yes) for each of the criteria, resulting in an overall score range of 0 to 18 for quantitative studies and 0 to 11 for qualitative studies. The scores were then converted into percentages. Once the team members completed quality assessments for their assigned articles, a separate meeting was held to discuss individual scores. Any discrepancies in scores were discussed, and scoring criteria were extensively reviewed to reach a consensus on a single score.

The research team established that only articles obtaining a total score of 90 or above were of sufficiently high quality to be included in this systematic review. Of the 33 articles that underwent the full extraction process, only 24 articles met this inclusion criterion of high quality.

### 2.4. Data Extraction and Outcome Classification

Data from the 24 articles were extracted and are presented in Table 1. Each article was broken down by authors, publication year, country, title, study aims, study design and instruments, sample and setting, findings and outcomes, and implications.

## 3. Results

### 3.1. Characteristics of Studies

Of the 24 articles included in this systematic review, seven were conducted in the United States, three in the United Kingdom/England, two in each country of China, Spain, Germany, and Japan, and one in each country of England and Japan, Singapore, Mexico, Portugal, Australia, and Canada. Twenty-three of the studies were quantitative, one was qualitative, and one was mixed methods.

Regarding studies of quantitative design, nine were longitudinal studies, while ten were described as cross-sectional studies. The studies were described as cross-lagged panel studies, sequentially designed, correlational studies, retrospective observational, prospective, explanatory sequential, and latent class analyses.

In terms of samples, of the seven studies conducted in the United States, four used data from the National Health and Aging Trends Study (NHATS), one was based on community-dwelling Korean immigrants, one was based on community-dwelling older adults in the Northeast United States, and one was based on data from the Centers for Medicare and Medicaid Services. Data were obtained from the Survey of Health, Aging, and Retirement in Europe (SHARE) for two studies conducted in Portugal and Spain. Four studies obtained data from the English Longitudinal Study of Aging (ELSA), while one study obtained data from the Population Health Index Survey. For studies conducted in Germany, one study used data from the German Aging Survey (DEAS), and one used data from the Activity and Function in the Elderly (ActiFE) Study. Studies conducted in Japan included one study with data obtained from the Japan Gerontological Evaluation Study (JAGES). In contrast, the second study used data from the Toyota Prevention Intervention for Cognitive Decline and Sarcopenia Trial. Of the two studies conducted in China, one study obtained data from the China Health and Retirement Longitudinal Study (CHARLS), while the second obtained data from the China Longitudinal Aging Social Survey (CLASS). The study conducted in Singapore obtained data from the Population Health Index. Of the two studies conducted in Australia, one study recruited older adults who were hospitalized, while the second study obtained data from the Australian Longitudinal Study on Women’s Health (ALSWH). Lastly, the study conducted in Mexico used data from the Mexican Health and Aging Study (MHAS).

Of the 23 quantitative studies, seven recruited participants aged 50 and older; three studies included participants 60 years and older, and thirteen had participants aged 65 years and older. There were no studies in which the sample participants were aged 24 to 49. Except for one study that included only women participants, 23 of the studies had mixed genders. Across all quantitative studies, there were 298,586 participants, which included 89 participants of the mixed methods study, from which 12 participants were enrolled in the qualitative arm of the study. The sample for the qualitative study consisted of 67 participants interviewed or enrolled in focus groups for a total sample of 298,653 adults aged 50 and older represented in this systematic review.

### 3.2. Summary of Included Studies

Based on the criteria for inclusion and the appraisal of the studies, the included studies were of high quality, examining the relationship between social isolation and variables representing physical health [refer to Table 1]. The studies included in this systematic review examined the relationship between social isolation and biomarkers, all-cause mortality, expectations of longevity, and frailty. In addition, other studies evaluated the relationship between social isolation and cognitive decline, sleep, and oral health. The relationships among social isolation, physical function/performance, and physical activity were also examined. Lastly, this review included studies that examined the relationship among social isolation, health, health behaviors, healthcare, and health-related quality of life, with some studies including the influence of selected demographic and clinical variables.

#### 3.2.1. Social Isolation and Biomarkers, All-Cause Mortality, Expectations of Longevity, and Frailty

Cudjoe et al. [33] examined the relationship between social isolation and biological markers of cytokine IL-6 and CRP in older populations. Their results indicated that social isolation is associated with higher levels of biological markers, informing the pathway between social isolation and morbidity and mortality in older adults. The implications were that biological markers may serve as an outcome measure for studies examining the effects of social isolation interventions. The relationship between social isolation and all-cause mortality among older Mexican adults was examined by Kammar-Garcia et al. [40]. The majority of the sample was female and 66 years of age. Forty-two percent reported loneliness, while fifty-three percent reported social isolation. The results indicated that only social isolation was associated with all-cause mortality and all individuals reporting social isolation presented with greater alterations in physical and mental health. Social isolation affected the search for appropriate medical treatment and adherence to medications. Social isolation increased the participants’ perception of threats and vulnerability, altered self-regulatory processes that influence physiologic functions, undermined sleep, and increased poor health behaviors, leading to increased morbidity and mortality. The authors emphasized that intervention programs to decrease social isolation by regaining and maintaining social activities and connectedness should be a priority of healthcare policymakers. 

Hajek and Konig [36], based on a national sample of individuals living in a shared household, reported that loneliness and social isolation were associated with lower expectations of longevity when adjusting for socio-economic and health-related variables, but the reported relationship was not affected by gender. Lower expectations of longevity were influenced by younger age, unemployment, the number of chronic health conditions, and lower self-rated health. The authors discussed how a low sense of purpose in life may lead to decreased subjective life expectancy. It was concluded that the relationship between social isolation and perception of longevity may become a self-fulfilling prophecy and lead to decreased health.

Ge et al. [12] reported on the relationship between social isolation and frailty. The researchers found that loneliness and lower social participation were associated with frailty. However, in contrast to other studies that reported a relationship between social isolation and frailty, this relationship was not supported. In addition, gender and socio-demographic variables of marital status and living arrangements were not associated with frailty. It was concluded that social isolation and loneliness had differentially associated relationships with frailty. Hayashi et al. [11] examined the impact of physical frailty and social isolation on falling in community-dwelling older adults. Their results indicated that, independently, social isolation was not associated with falls; however, social isolation and frailty, in combination, were associated with falls. This conclusion suggested the necessity for further studies to explore the relationship between social isolation and frailty and to elucidate whether falls are more prevalent among individuals who were physically frail before experiencing social isolation or vice versa.

#### 3.2.2. Social Isolation and Cognitive Decline, Sleep, and Oral Health

The relationship between social isolation and cognitive decline/health was examined by Guo, Luo, Gao, and Yu [35], who reported that higher levels of social isolation were associated with decreases in episodic memory over time for individuals with depressive symptoms, as well as 4-year cognitive decline, relative to women, not men. The relationship between social isolation and episodic memory was explained as social isolation induces stress and causes cognitive impairments through decreased connectivity and plasticity of the prefrontal cortex, resulting in memory impairments. Secondly, it was thought that social isolation and depressive symptoms may synergistically increase the release of glucocorticoids, resulting in neurodegeneration of the hippocampus related to memory. Jang et al. [8] demonstrated social isolation and cognitive impairment were positively associated with older Korean Americans. Loneliness was found to be a mediator in the relationship between social isolation and subjective cognitive impairment, but not objective cognitive impairment. Furthermore, social isolation was found to pose a significant risk for both subjective and objective cognitive impairment, even after controlling for socio-demographic variables, immigration-related characteristics, chronic medical conditions, and depressive symptoms. The authors emphasized that their results provided clinical insight into the care of immigrant populations and that interventions are needed to reduce loneliness and social isolation to prevent early cognitive decline. Silberman-Beltramella et al. [46], based on older adults in Spain, found social isolation, related to social relation characteristics of social network size and satisfaction, was associated with physical and emotional health, cognitive, sensory abilities, and functional ability. The implications identified the importance of developing and reinforcing social support networks for older adults and home care strategies, including the role of liaison nurses, in managing available resources and encouraging family involvement to enhance network size and increase satisfaction with social relations. 

In examining the effect of social isolation and sleep, Zhang, Lin, Chen, and Schuzhuo [48] reported that social isolation was an independent risk factor for sleep difficulties. In addition, social isolation was found to be positively associated with chronic diseases and pain, as well as higher levels of depression. The authors concluded that future research is needed to explore how social isolation related to different types of relationships affects sleep and how such an understanding may be important to preventing sleep problems. It was recommended that policymakers support interventions to enhance social connectedness from family and friends to broader social networks. 

Koyama et al. [42] examined the differences between oral health status and social isolation among older people from Japan and England. More Japanese elders were socially isolated than English, but fewer Japanese were edentulous. In both countries, poorer oral health was associated with social isolation. Those who lacked teeth had higher social isolation scores in England as compared with those in Japan. The conclusion was that more studies are needed to examine the effectiveness of interventions to promote oral health and reduce tooth loss, which may reduce the risk of social isolation in older adults. 

#### 3.2.3. Social Isolation, Physical Function/Performance, and Physical Activity

The relationship between social isolation and physical function among older adults was examined in two studies conducted by del Pozo et al. in 2021 [9,34]. Their findings demonstrated that greater levels of social isolation were associated with lower physical function over a period of five years. An increase in physical function decreased social isolation over three years. The bidirectional results suggested that interventions that increase physical function or reduce social isolation are needed, which may inform the optimal timing for the re-delivery of interventions to maintain their effectiveness. Furthermore, there was a substantial moderating effect of age between social isolation and physical functioning, with social isolation accelerating a decline in physical functioning associated with aging. The authors proposed that social isolation decreases physical functioning as it is associated with the dysregulation of inflammatory and neuroendocrine processes. Public health policies are needed to promote high-quality social relations among older adults and support the development of programs that result in scheduled personal contact with existing or new members of the social network. 

Imamura et al. [38] studied the relationship between social isolation and the future decline in physical performance/function in community-dwelling elderly adults. Based on a one-year longitudinal study, it was reported that social isolation at baseline was significantly associated with the future Time Up and Go (TUG) Test, which is a measure of the functional ability to get up and walk, determined at a one-year follow-up. Social isolation was not associated with a decline in other physical functioning, such as muscle strength and physical performance measuring hand-grip strength, knee extensor strength, or 5-minute walking time. The authors concluded that social isolation and TUG are risk factors for disability. Furthermore, over time, social isolation may be associated with a decline in physical performance by limiting healthy lifestyles and the frequency of interactions with others. 

Several studies reported the association between social isolation and physical activity. Deleru Matos et al. [15] reported that highly socially isolated adults over the age of 50 are more prone to being physically inactive as well as having inadequate diets in terms of the consumption of fruits and vegetables. In addition, high social isolation was associated with lower physical health, greater depression, and more doctor visits. The majority of those who were highly socially isolated were individuals over the age of 70, female, less educated, and low income. It was concluded that public and social health policies should promote opportunities for social interactions, physical activity, and a balanced diet.

Herbolsheimer et al. [37] studied the relationship between social isolation and indoor and outdoor physical activity in community-dwelling older adults. Their results indicated that low levels of physical activity were associated with social isolation. Individuals who were socially isolated from family reported low indoor physical activity, while those who were socially isolated from friends and neighbors reported low outdoor physical activity, including meeting people for shopping, walking, or attending cultural events. They concluded that future research is needed for a more nuanced assessment of non-kin networks and locations in which physical activities occur, as well as learning about how different kinds of physical activities and perceived social isolation can improve physical activity programs. 

Robbins et al. [29], based on community-dwelling older adults recently discharged from the hospital, explored the relationship among physical capacity, measured by a disability scale, physical activity (recreation vs. household-based), and social isolation. Between baseline and three months post-hospital discharge, decreased social isolation was related to improved physical activity. From baseline going forward 6 months, an increased number of friends seen was associated with increased physical capacity and physical activity. It was reported that the more contact with relatives, the greater the household-based physical activity. The findings supported the need to investigate interventions that target physical activity and physical capacity among socially isolated individuals during two weeks post-hospitalization.

Schrempft, Jackowska, Hamer, and Steptoe [25] demonstrated the association among social isolation, loneliness, and objective physical activity in older men and women. Independent of age, gender, socio-economic status, marital status, smoking, alcohol consumption, and self-rated health, the results indicated that physical activity over a 24-h period was lower in socially isolated individuals. Sedentary behavior during the day and evening was greater in socially isolated individuals. Physical activity was greater on weekdays than on weekends, but the levels of social isolation were comparable. The findings suggest differences in physical activity may contribute to poorer health. 

#### 3.2.4. Social Isolation and Health, Health Behaviors, Self-Care, and Health-Related Quality of Life

Smith and Victor [47] explored topologies of loneliness, living alone, and social isolation related to physical and emotional health. Groups reporting social isolation based on household composition, social activities, and communication with family and friends and loneliness reported poorer physical and emotional health after controlling for confounding variables for those who reported high loneliness and moderate social isolation and lived alone. Living alone was found to be conceptually different from loneliness and social isolation. Poor health was also found to be a risk factor. Those with high levels of loneliness or social isolation also reported greater depression. The conclusion was that social isolation and loneliness are differentiated concepts and poor health may lead to loneliness or social isolation. Therefore, the direction of causality should be further explored, as well as the severity of loneliness and social isolation. Identifying groups with differing characteristics of loneliness and social isolation requires data-driven methodologies to understand how these concepts are related to health.

Pohl, Bell, Tancredi, and Woods [14] were interested in examining the prevalence of social isolation in caregivers, as well as the associations between social isolation and health. Less social isolation was reported, with better self-reported health. Demographic factors associated with higher social isolation included being Black, non-Hispanic, or Hispanic, having less than a college education, and being family caregivers. Lack of community participation was associated with less self-reported health, particularly among younger caregivers. The authors concluded that when conducting caregiver health assessments, social isolation should be evaluated, along with the assessment of caregivers’ participation in their communities and disconnection from friends.

To develop an instrument to measure social isolation, Pohl, Cochrane, Schepp, and Wood [44] (2017) conducted a study based on a sample of older adults. Socially isolated participants were likely to be older, white, non-Hispanic, and live alone. Those who were socially isolated had higher mean depression scores and lower mean well-being scores. 

In examining the relationship between social isolation and health behaviors, Kobayashi and Steptoe [41] reported that individuals aged 50 years and older who were socially isolated consistently demonstrated lower levels of moderate to vigorous physical activity and ate less than five fruits or vegetables per day than those who were not socially isolated. The authors concluded that in older adults, social isolation leads to non-engagement in healthy behaviors. Therefore, social isolation affects health outcomes and may increase mortality rates through behavioral pathways, warranting psycho-social interventions to reduce social isolation.

LeBlanc, Chiodo, and Jacelon [43] conducted a mixed methods study to determine how social networks influence therapeutic self-care behaviors and health among older adults with multiple chronic conditions. Their results, based on the quantitative arm of the study, indicated that positive social relationships influenced mental health but not physical health. Social network ties were not associated with symptoms, general health management, or administration of medication. However, there was a positive relationship between social network ties and self-care activities. It was concluded that certain aspects of self-care were more socially influenced, while other aspects were more privately managed. Social support was found to influence self-care and health. It was proposed that social support is beneficial to influence self-care through affective processes that reduce pain, anxiety, and depression. The qualitative arm of the study revealed that for older adults with chronic illness, the size of the social network decreased over a participant’s life span because of the loss of members, choices to limit relationships, and physical and emotional health. A second theme was that in dealing with a fluctuating health status, asking for help was described as a process of learning. Asking for help had cultural implications when independence was a value. Lastly, participants expressed the importance of using phone technology for social connection and obtaining not only psycho-social support but tangible help. The authors concluded that the telephone is a basic tool that can be used to ask for social support and promote relationships that influence health. 

As the single qualitative study included in this systematic review, Salma and Salami [45] conducted a study to understand the experience of social isolation and loneliness in Muslim immigrant older adults in Canada. The themes related to social isolation included ageism, racism, and sexism. Though the participants felt connected with their children and grandchildren, they did not feel connected with peers and did not feel that there were welcoming public and private spaces. Older Muslim women also felt gender-based discrimination from within their own Muslim community. The participants expressed the need for welcoming and safe spaces where older adults could interact with people from other cultures and religions and sought programs that combine socialization, physical activity, and prayer. The results highlighted the need for interventions that promote personal and community-level agency, including volunteer and work opportunities.

Freak-Poli et al. [49] provided additional evidence regarding the influence of social isolation, social support, and loneliness as being independently associated with health-related quality of life of women aged 70 and older. The majority reported being socially isolated, yet only 9% and 14% had low social support or were lonely, respectively. There were strong negative relationships between social isolation, low social support, and loneliness and physical and emotional health and health-related quality.

## 4. Discussion and Implications

The majority of studies identified as high quality for inclusion in this systematic review were based on large international data sets representing 298,653 older adults. Nine of the quantitative studies were of a longitudinal design, while the majority were correlational. Only recently, post the COVID-19 pandemic, has the phenomenon of social isolation come to the forefront of clinical care for adults. To understand this phenomenon, it is expected that research studies begin with descriptive, exploratory studies to examine the relationship between social isolation and physical health and potential mediating or moderating variables. To advance the science regarding social isolation, this systematic review focused on social isolation and various aspects of physical health for the purpose of developing and testing interventions to reduce social isolation and provide evidence to inform public health policies and community programs to reduce social isolation and enhance the health of older adults. This study will assist in the future development of an extant conceptual framework regarding social isolation and physical health and a conceptual diagram of the related concepts validated by this systematic review. Suggestions emerging from this framework must be further studied through quantitative, qualitative, and mixed methods research as this will provide evidence to guide the development and future testing of interventions to reduce social isolation in adults.

Based on the findings of this systematic review, we begin with the discussion of social isolation and biomarkers, all-cause mortality, expectations of longevity, and frailty. Based on the studies examining social isolation and biomarkers, it was evident that severe social isolation was significantly correlated with inflammatory markers of IL-6 and CRP levels. Inflammation is the underlying cause of many diseases, including cancer and heart disease, and informs the pathway of social isolation, morbidity, and mortality. It is suggested that inflammatory biomarkers serve as outcome measures for future clinical and social interventions. In addition, inflammatory markers, such as TNF and Il-1B, which are more specific than IL-6 and CRP levels, should be measured in longitudinal studies [33]. Kammar-Garcia and colleagues [40] substantiated the relationship between social isolation and all-cause mortality, proposing that social isolation increases feelings of threat and vulnerability, which affect self-regulatory and physiologic processes that influence physical function and sleep and increase unhealthy behaviors. Importantly, social isolation also decreases perceptions of longevity, becoming a self-fulfilling prophecy [36], with possible ultimate effects on morbidity and mortality. Recognizing the effects of differing yet similar concepts of loneliness, social participation, and social isolation [12], social participation, often measured as an aspect of social isolation, was found to be associated with the frailty of adults, particularly older adults. Ge et al. [12] alerted health professionals that risk factors, such as loss of a loved one, chronic illness, living alone, and declined mobility, as well as hearing and vision loss, exacerbate social isolation, lowering social participation and increasing frailty. Furthermore, the combination of social isolation and frailty was associated with falls [11], which was also documented in the literature as a significant contributor to morbidity and mortality of older adults [50,51]. 

The impact of social isolation on cognitive decline, sleep, and oral health was also examined. A natural aspect of aging is cognitive decline, yet the concern is that social isolation increases memory loss, particularly in women who are also diagnosed with depression [36]. Again, the postulated pathway is that the stress of social isolation may decrease connectivity and plasticity in the pre-frontal cortex, resulting in memory impairment. With a synergistic effect of depression, social isolation may also lead to the release of glucocorticosteroids that lead to the degeneration of the hippocampus, which is also associated with memory [35]. Besides issues with memory, social isolation was found to affect sleep, which is a basic human need and a vital aspect of overall health [52]. Of social significance, there was a relationship between social isolation and oral health, indicating that those who were missing teeth had greater social isolation [42]. As reducing social isolation is important to physical health outcomes, it was suggested that interventions to improve oral health may result in less social isolation.

The relationship between social isolation and physical function/performance and physical activity requires discussion. Based on the work of del Pozo Cruz and colleagues [9,34], it was found that a one unit increase in physical performance was associated with a decrease in social isolation. In contrast, an increase in social isolation was associated with a decrease in physical performance. The authors further substantiated the physiological mechanisms associated with social isolation, suggesting that social isolation decreases PHYSICAL HEALTH because of a dysregulation of inflammatory and neuro-endocrine processes. It was suggested that policies should focus on interventions that increase social connectedness and participation, such as friendship clubs, and community partnership interventions, such as home visits by allied health professionals and community members. Furthermore, policies that optimize the timing, recurrence, and dosing of social isolation interventions need to be examined and instituted. Several studies also reported the association between social isolation and physical activity, including indoor and outdoor activities [37,38]. It was recommended that social support be provided to assist adults, particularly older adults, with activities of daily living and companionship for shared activities that increase a sense of well-being [38]. Additionally, future research is needed for a more nuanced assessment of non-family networks and locations in which physical activity can occur. Learning more about how different kinds of physical activity are associated with a decrease in social isolation would be informative to the development of physical activity programs [47], not only for adults and older adults but also for those who are post-hospitalization [29]. 

Several studies revealed the relationship between social isolation and overall health, as well as the relationship with health behaviors, self-care, and health-related quality of life. Overall, it was documented that higher social isolation is associated with poorer physical health, but the authors emphasize that more research is needed to validate the direction of causality [37]. Does poor physical health lead to social isolation or the reverse? Smith and Victor [47] suggested that studies should explore the differing characteristics of social isolation and loneliness to determine how these concepts are related to health. Furthermore, it was emphasized by Pohl et al. [44] that clinical assessments of older adults and caregiver populations should include various aspects of social isolation, using qualitative or quantitative methodologies, to understand social isolation and health more fully and inform potential interventions to improve social isolation. The work by Kobayashi et al. [41] emphasized that social isolation is associated with certain healthy behaviors, and those who are socially isolated have lower levels of physical activity and less consumption of the recommended number of fruits and vegetables daily. These results reinforce the importance of behavioral pathways associated with social isolation and the significance of psycho-social interventions in reducing social isolation. Equally important are the findings regarding the relationship between social isolation and self-care. Le Blanc et al. [43] found that social network ties and self-care are associated, and certain aspects of self-care are more socially influenced, while others are private. Social support from family, friends, and healthcare providers can encourage older adults to engage in healthy behaviors. In examining social isolation and health-related quality of life, it was documented that social isolation has a significant impact on how individuals perceive their quality of life overall [49].

Finally, the introduction to this article speaks to the biological, behavioral, and psychological pathways of social isolation [22]. The studies presented in this review reinforce health outcomes associated with biological and behavioral pathways. A future article regarding social isolation and psychological health by this research team is expected to inform the psychological pathway. The biological pathway of social isolation gives rise to positive associations among inflammatory biomarkers, all-cause mortality, expectations of longevity, and frailty, as well as physical function/performance, physical activity, and diet. The behavioral pathway underpins the relationship between social isolation and health, health behaviors, self-care, and health-related quality of life. Several studies have either statistically controlled potential confounding variables, primarily demographic or clinical, or have reported on the relationship between social isolation and physical health. In the introduction of this article, risk factors related to social isolation in adults were identified [20,29,30]. With substantiation of the relationship between social isolation and physical health, further studies are needed to examine the relationship between risk factors and increasing social isolation [29]. These risk factors may either be considered constant, unmodifiable, or potentially modifiable when considering the development of interventions to reduce social isolation or modify, in a positive way, the relationships between social isolation and the variables examined in this review.

## 5. Strengths and Limitations

As a strength, this systematic review was a response to the call from the National Institutes of Health to examine the relationship between social isolation and health. The search process and appraisal of the literature were assisted by the use of Covidence, which limited study biases. This review included high-quality studies that were identified through a very diligent review of articles, in which three researchers critiqued and rated each article and made the decision to only include studies with a score of 90 or above. All members of the research team were highly engaged in data collection and data analysis and participated, through the use of Google Docs, in editing this manuscript.

A further strength of this study was that it represents diverse populations worldwide. Future research can provide value by comparing the experience of social isolation and health within various geographic locations within the United States with other countries. In addition, mixed methods studies and qualitative studies can strengthen the conceptualization of social isolation and health and be of significant value in informing the development of interventions to reduce social isolation and improve health. 

A limitation of this study was the narrowing of the scope to social isolation and physical health given the large number of published studies. The future plan is to conduct a second systematic review focused on social isolation and psychological/emotional health. This systematic review also excluded other systematic reviews and studies regarding COVID-19, as it was a unique worldwide pandemic that demanded social isolation from all sectors of society. In addition, studies regarding social isolation in veterans were excluded as the veterans represent a special population. Although the inclusion criteria in this systematic review included adults aged 24 and older, the study population was skewed to adults aged 50 years and older. This indicates that research regarding social isolation in younger adults (ages 18 to 23) is warranted, particularly since the use of social media has limited face-to-face social interactions and has resulted in an increase in health issues across populations within the United States and abroad. 

Furthermore, this systematic review identified studies that link the inflammatory processes associated with social isolation and disease, such as heart disease and other chronic conditions. However, given the search parameters, no studies were identified that reported directly on the relationship between social isolation and the incidence of acute or chronic diseases. In addition, research studies, specifically biological studies, did not report brain imaging and genomics related to social isolation. A further search regarding these primary studies would be important. Furthermore, as a systematic review, the majority of studies included in this systematic review were correlational in design, with few being longitudinal studies. Future longitudinal research is needed, along with the need to move from descriptive, exploratory studies to proposed intervention studies to test ways of reducing social isolation and improving health across the lifespan. Lastly, an additional limitation was that this systematic review was conducted and submitted for publication prior to registering the study as a systematic review as requested by the journal. In an attempt to register the systematic review, the authors learned that part of the registration process included identifying any other systematic reviews on the same topic. However, given that this study was completed, it was no longer eligible for registration.

## 6. Conclusions

The researchers represented in this systematic review have sounded a call for future studies regarding social isolation, emphasizing the need to monitor its effect over time on physical, emotional, and socio-cultural health across worldwide populations. The emphasis is on the need for biological and behavioral interventions to reduce social isolation and improve physical health. This is critically important as social isolation is a public health crisis, with negative outcomes that influence the health of adults in addition to having family, community, and societal implications.

## Figures and Tables

**Table 1:** Studies Included in the Systematic Review.

	Authors, Year, Country	Article Title	Study Aim	Study Design and Instruments	Sample and Setting	Findings and Outcomes	Implications
1	del Pozo Cruz, B., et al. (2021) USA [9]	Bidirectional and dynamic relationships between social isolation (SI) and physical functioning (PF) among older adults: A cross-lagged panel model of US national survey data	To identify the bidirectional associations between social isolation and physical functioning in older adults and the associated temporal dynamics.	A general cross-lagged panel model was used to analyze the Social Isolation Index and Short Physical Performance Battery.	The sample was drawn from nine waves of panel data from the National Health and Aging Trends Study (NHATS) that sampled 12,427 U.S. adults aged 65 or older between 2011 and 2019	The findings indicated that greater levels of SI at a given time point were associated with lower scores in PF in the future. A second key study finding was the identification of strong temporal dynamics in which an increase in social isolation reduced physical functioning over a period of five years, while an increase in physical functioning decreased social isolation for a period of three years. These novel insights suggest that interventions to enhance SI and/or PF need to be periodically re-delivered to maintain their effectiveness.	The study confirms the existence of statistically significant bidirectional associations between SI and PF among older adults in the United States. Because the effect of SI dominate, the findings indicate that public health strategies to promote successful aging should prioritize interventions that enrich older adults’ social networks. Furthermore, the effects’ time horizons yielded by the model serve as fruitful avenues to calculate the optimal timing for the re-delivery of preventive interventions.
2	Cudjoe, T.K.M., et al. (2022) USA [33]	Getting under the skin: Social isolation and biological markers in the National Health and Aging Trends Study	To examine the relationship between social isolation and biological markers cytokine IL-6 and CRP in older populations.	A multivariable linear regression model was used to examine the association between the study variables and socio-demographic variables, as well as smoking status, BMI, comorbidity, depressive symptoms, frailty, and dementia. Social isolation was measured by the Berkman–Syme Social Network Index and the biomarkers of IL-C and CRP were measured by dried blood spot samples.	The sample was from Round 7 (2017) data from the NHATS of 4648 U.S. Medicare beneficiaries aged 65+.	Social isolation was associated with higher levels of biological markers (IL-6 and CRP). Findings informed the pathway between social isolation and morbidity and mortality among older adults. IL-6 or CRP could be a proximal outcome measure for future clinical and social interventions that seek to alter the trajectory of social isolation and its associated health outcomes.	These findings are important because clinical and social interventions to address social isolation among older adults may influence the studied biological processes and their potentially negative effects. Furthermore, biomarkers may serve as an important outcome measure for social isolation interventions.
3	Delerue Matos, A., et al. (2021) Portugal [15]	Social isolation, physical inactivity and inadequate diet among European middle-aged and older adults	To examine the relationship between social isolation and health risk behaviors, such as inadequate diet and physical activity, among middle and older age European adults.	Cross-sectional study involving a two-group comparison of highly socially isolated individuals and low socially isolated individuals. Regressions by country were performed to examine the relationship between social isolation and physical inactivity and inadequate diet. Age, gender, education, income, excessive alcohol consumption, smoking, number of doctors’ appointments and physical and mental health were co-variates.	Data from the Survey of Health, Aging, and Retirement in Europe (SHARE) of 67,173 adults aged 50+ from 17 European countries.	Highly socially isolated individuals were 70 years or older, female, less educated, and of low income. Women were more isolated because of greater involvement in housework and caregiving responsibilities. Higher levels of social isolation were associated with less physical health, greater depression, and doctor visits, but lower alcohol consumption and smoking. Highly socially isolated European middle-aged and older adults were more prone to be physically inactive and to have an inadequate diet in terms of daily consumption of fruit and vegetables on a daily basis. The reduced social integration, social support, and companionship of the highly socially isolated individuals may explain this association.	The results reinforce the need for public social and health policies targeted towards European socially isolated middle-aged and older adults. Policies should counter social isolation by creating opportunities for social interaction or, at least, should reduce the effect of social isolation through social support capable of promoting opportunities for engaging in physical activity and having a balanced diet.
4	del Pozo-Cruz, B., et al., (2021) USA [34]	Impact of social isolation on physical functioning among older adults: A 9-year longitudinal study of a U.S.-representative sample	To examine the relationship between social isolation and physical functioning using a longitudinal research design.	Fixed-affect regression model analysis on longitudinal data from a national survey. Social isolation was measured by the Social Isolation Index and physical functioning was measured by the Short Physical Performance Battery.	The sample was from nine waves of panel data from the NHATS study of 12,427 U.S. adults aged 65+ from 2011 to 2019.	A key finding was that social isolation was associated with poorer physical functioning. There was a substantial moderating effect of age in the relationship between social isolation and physical functioning. Social isolation accelerated the decline in physical functioning associated with aging.	The findings add to a growing body of evidence demonstrating the negative consequences of social isolation, specifically acceleration of aging associated with physical decline. There is a negative effect of social isolation on physical function through dysregulation of several psychobiological responses, including inflammatory or neuroendocrine processes. Study findings indicate that public health interventions should focus on social environments and develop policies that promote social contact and high-quality social relations among older adults. This may occur through skill development programs, scheduled personal contact with existing or new social network members, and animal-assisted interventions.
5	Ge, L., et al., (2022) Singapore [12]	Associations of social isolation, social participation, and loneliness with frailty in older adults in Singapore: A panel data analysis	To examine the longitudinal associations among social isolation, social participation, and loneliness with the level of frailty among community-dwelling older adults. The study used panel data from the Population Health Index (PHI) Survey and explored the moderating effect of gender. Frailty is a common geriatric syndrome characterized by cumulative presentation of clinically identifiable somatic deficits, decreased physiological reserve, and heightened vulnerability to stressors.	Longitudinal study using the Population Health Index (PHI) Survey including measures of frailty (CFSI-7), social isolation (Lubben Social Network Scale), social participation (Social role domain of the Late-Life Function and Disability Instrument), and loneliness (UCLA Loneliness Scale). Co-variates included the socio-demographic data on age, marital status, employment status, living arrangement, financial status, smoking status, alcohol misuse, number of diagnosed chronic conditions, medications, and functional and nutritional status.	The study included 606 participants aged 60 years and above from the longitudinal PHI Survey conducted in Singapore.	Social isolation and social participation were moderately associated with each other and weakly associated with loneliness. An increase in social participation was associated with a lower level of frailty. Feeling lonely was associated with a higher level of frailty. Social isolation was not associated with frailty, which contrasted the results of earlier studies. Gender did not have moderating effect on these associations. In contrast to other studies, changes in other socio-demographic characteristics, such as marital status or living arrangement, were not associated with frailty.	The study shows that social isolation and loneliness have a differential longitudinal association with the level of frailty among community-dwelling older adults. Social participation and feeling of loneliness are independently associated with higher level of frailty in older adults and gender does not moderate the associations.
6	Guo, L., et al., (2021) China [35]	Social isolation and cognitive decline among older adults with depressive symptoms: Prospective findings from the China Health and Retirement Longitudinal Study	To examine the association between social isolation and cognitive decline among older adults with depressive symptoms in a non-Western country.	Longitudinal study examining depressive symptom measured by the Center for Epidemiological Studies Depression Scale and social isolation assessed based on items including marital status, residence, contact with children, and social activity. Cognitive function was measured by an episodic memory measurement to assess immediate and delayed recall and mental status questions from the Telephone Interview of Cognitive Status (TICS) battery.	The data were obtained from the China Health and Retirement Longitudinal Study (CHARLS), a nationally representative longitudinal survey of the middle-aged and elderly population (n = 2507) (mean age 61; male 41%) in China.	A higher level of social isolation was significantly associated with decreases in episodic memory over time for older adults with depressive symptoms and 4-year cognitive decline. The interaction between gender and social isolation for predicting episodic memory, and further analysis revealed that depressed women were more vulnerable to the impact of social isolation. Thus, compared with men, women who had experienced social isolation may have a higher risk of memory decline. A biological pathway of social isolation related to inducing stress. The association between social isolation and episodic memory in depressed older adults may be related decreased connectivity and plasticity of the prefrontal cortex and a possible synergistic effect on the increased release of glucocorticoids that may result in neurodegeneration of the hippocampus related to memory. There was no association between social isolation and mental status in depressed older adults in contrast to the results of previous studies.	These findings expand our knowledge about the association between social isolation and cognitive decline in non-Western depressed populations. Further studies are warranted to clarify how social isolation affects domain-specific cognitive capacity among people with depressive symptoms.
7	Hajek, A. and Konig, H. (2021) Germany [36]	Do lonely and socially isolated individuals think they die earlier? The link between loneliness, social isolation and expectations of longevity based on a nationally representative sample	To examine the relationship among loneliness, social isolation, and expectations of longevity in a nationally representative sample of individuals middle aged or older.	Cohort sequential designed, cross-sectional study based on a national probability sampling using multiple regression analyses. Social isolation was assessed by the Bude and Lantermann Scale, while loneliness was measured by the De Jong Gierveld Loneliness Scale. Expected longevity in years was measured as a single question.	A sample of 4857 individuals enrolled in the German Aging Survey (DEAS) sixth wave (year 2017). Participants were 50 years or older living in a private household.	Loneliness and social isolation were associated with lower expectations of longevity when adjusting for various socioeconomic and health related covariates. The association between social isolation and expectations of longevity was not affected by gender. Lower expectations of longevity were associated with younger age, being retired, not being employed, worse self-related health, and the number of chronic illnesses. The findings may indicate that individuals with high levels of social isolation may also have a low purpose in life with decreased subjective life expectancy.	Future studies based on longitudinal data are required to gain further insights. Knowledge about the association between social isolation and perception of longevity is important because low expectations of longevity can become a self-fulfilling prophecy and lead to decreased health.
8	Hayashi, T., et al., (2020) Japan [11]	Combined impact of physical frailty and social isolation on rate of falls in older adults	To examine the impact of the combination of physical frailty and social isolation on falling in community-dwelling older adults using logistic regression.	A cross-sectional study of data obtained at registration in a randomized control trial analyzed cross-sectional baseline data from the TOPICS (Toyota Prevention Intervention for Cognitive Decline and Sarcopenia) Trial. Social isolation was measured by the Lubben Social Network Scale. Falls were measured as the number of times in the past year the respondent came to rest on the ground. Frailty assessment was measured by slowness, weakness, exhaustion, low activity, and weight loss. The Frailty Index was a battery of neuropsychological tests, physical assessments, and blood tests. Covariates included age, sex, family status, walking aids, body mass index, educational years, medical conditions, number of medications, physical function, depressive symptoms, and cognitive function.	A community-based study of 380 community-dwelling older adults recruited from Toyota, Japan. Participants were divided into four groups depending on non-frail and pre-frail/frail status, based on Fried frailty criteria and social isolation, based on the Lubben Social Network Scale, as not socially isolated or socially isolated. The incidence of multiple falls over the past year were compared among groups.	Physical frailty and social isolation were not independently associated with falling, but physical frailty and social isolation combined was significantly associated with falling as compared with the robust group after controlling for confounding factors. The findings support the assertion that the coexistence of physical frailty and social isolation were associated with falling in older adults. No differences in the severity of frailty status were found between the physical frailty and social isolation and the physical frailty groups.	Further studies are needed to determine the bidirectionality of the relationship between social isolation and falls. Further studies are required to clarify the relationship between physical frailty and social isolation and the degree of physical frailty.
9	Herbolsheimer, F., et al., (2017) Germany [37]	Relationship between social isolation and indoor and outdoor physical activity in community-dwelling older adults in Germany: Findings from the ActiFE Study	To better understand the relationship between physical activity and social isolation in old age, the study investigated the following: (a) whether older adults’ (objectively assessed) physical activity levels are differently associated with two sources of social isolation (i.e., friend/neighbors and family) and (b) whether indoor and outdoor physical activity is differently related to social isolation.	A cohort, cross-sectional study measuring social isolation using the Lubben Social Network Scale (LSNS-6) and physical activity measured by an accelerometer (activPAL). Participants kept a contemporary physical activity diary to report outdoor physical activity timeframes.	A sample of 1162 community-dwelling older persons (mean age = 75.6; SD = 6.6) from the greater area of Ulm in Germany was recruited in the Activity and Function in the Elderly (ActiFE) study. Participants aged between 65 and 90 were randomly selected.	Low levels of physical activity were associated with perceived social isolation. Low indoor physical activity was associated with being socially isolated from family, and low outdoor physical activity was associated with being socially isolated from friends and neighbors (*p* = 0.012). Low physical activity in outdoor locations was strongly associated with perceived social isolation from friends and neighbors. Diary data revealed that social isolation from family and friends was related to less outdoor physical activity involving meeting people or visiting cultural events in comparison with non-isolated individuals. This substantiated the claim that differences in outdoor physical activity were associated with social relations. Furthermore, social contacts were also closely connected to other outdoor activities, such as shopping or going for a walk.	These findings suggest the need for a more nuanced assessment of non-kin networks and a differentiated analysis of the locations in which physical activity is performed. Further studies are needed to determine how social isolation affects every day physical activity. A greater understanding of the mechanisms of the association between different kinds of physical activity and perceived social isolation can be used to create and improve physical activity programs. Such programs might be most beneficial if they target friend, neighbor, and peer networks as a means to improve individual physical activity.
10	Imamura, K., et al., (2022) Japan [38]	Social isolation is associated with future decline of physical performance in community-dwelling older adults: A 1-year longitudinal study	To examine whether social isolation is associated with a future decline in physical function in older people	Longitudinal study that examined whether social isolation was associated with future decline in muscle strength and physical performance in community-dwelling older people. Social isolation was measured using the Lubben Social Network Scale and physical function was measured by handgrip strength, knee extensor strength, usual walking time, and the Timed Up and Go test, with analysis using the logistic regression adjusting for confounding variables.	The participants were 166 community-dwelling older people. The participants were aged 65 years and over, lived in the community, and were recruited from participants in health check-ups for geriatric syndrome organized in 2016 by a university research team and a community sports facility in Japan. The mean age of participants was 73.3, and 67.5% were women.	Based on a one-year follow-up survey, the results showed social isolation at baseline was significantly associated with future Timed Up and Go (TUG) decline in well-functioning older adults after adjusting for potential confounding variables. Social isolation was not associated with a decline in usual walking time, handgrip strength, or knee extensor strength. Almost all participants could perform independent activities of daily living (IADL).	The results indicated that assessment of social isolation may be necessary to assess the risk of physical performance decline. Even in well-functioning older adults, social isolation is a risk factor in the decline of physical performance.
11	Jang, Y., et al., (2021) USA [39]	Cognitive health risks posed by social isolation and loneliness in older Korean Americans	To examine the associations among social isolation, loneliness, and objective and subjective measures of cognitive impairment in older Korean Americans.	Data from the Study of Older Korean Americans (SOKA) was used. Data included the SOKA questionnaire, the Lubben Social Network Scale-6; the short-form UCLA Loneliness Scale, and objective and subjective measures of cognitive impairment measured by the Mini-Mental State Examination and a single-item self-rating of cognitive health. Covariates included age, gender, marital status, education, perceived financial status, and length of stay in the U.S., as well as chronic medical conditions and depressive symptom using the Patient Health Questionnaire 2.	Data from a multi-state survey of Koren immigrants aged 60 and older (n = 2061); the mean age was 73.2 with 67% being female and over 60% being married.	Social isolation and cognitive impairment were positively associated, but loneliness was not. Subjective cognitive impairment was statistically associated with social isolation and loneliness. Loneliness was found to be a mediator in the association between social isolation and subjective cognitive impairment, but not with objective cognitive impairment. In multivariate analyses, social isolation was found to pose a significant risk to both objective and subjective cognitive impairment after controlling for the effects of sociodemographic and immigration-related characteristics, chronic medical conditions, and depressive symptoms. The link between social isolation and objective impairment was unaffected by loneliness.	In further analyses, these findings imply that different dimensions of social disconnectedness hold different implications for objective and subjective cognitive health. The robust impact of social isolation on objective cognitive impairment reflects the critical role of the structural aspect of social relationships as a potential source of cognitive reserve. On the other hand, loneliness plays a critical role in predicting subjective cognitive impairment, and its entry into the analytic model made the effect of social isolation non-significant. The findings add to the literature suggesting that social isolation and loneliness may have differential cognitive health consequences and emphasize the need for nuanced assessments. Consequently, these findings provide clinical insights for the care of older immigrants; that is, interventions to reduce feelings of loneliness might be a fruitful strategy for managing or preventing early cognitive decline, possibly by engaging participants in socially meaningful and cognitively stimulating activities.
12	Kammar-Garcia, A., et al., (2023) Mexico [40]	Association of loneliness and social isolation with all-cause mortality among older Mexican adults in the Mexican health and aging study: A retrospective observational study	To analyze the longitudinal association among loneliness, social isolation, and their interactions with the all-cause mortality among older adults in Mexico.	A retrospective observational study based on a cohort from the Mexican Health and Aging Study (MHAS) in the 2015 and 2018 waves. Loneliness was measured using the Revised UCLA Loneliness Scale and social isolation was measured using the Berkman and Syme Social Network Index. All-cause mortality during the three-year follow-up period were based on face-to-face interviews of participants. Multi-variate adjustment for covariates of demographic data, clinical variables, psychological characteristics, cognitive status, and lifestyle characteristics.	Mexican adults older than 50 years were included in the study. The final sample included 11,713 participants. Participants were classified according to their level of loneliness and the presence of social isolation.	Of the sample, the mean age was 66.6 years, and the majority (58.2%) were female. The incidence of all-cause mortality in 3 years of follow-up was 6%. Forty-two percent were lonely, and fifty-three percent were socially isolated. Based on multivariable adjustment, only social isolation was associated with all-cause mortality, and the interaction between loneliness and social isolation was not associated with all-cause mortality. Individuals with any degree of loneliness or social isolation presented with greater alterations in physical and mental health. Socially isolated or lonely adults may search for appropriate medical treatment and adherence to medications. Socially isolated adults have an increased perception of threats and vulnerability. This hypervigilance may alter psychological self-regulatory processes that influence physiologic functions, undermine sleep quality, and increase unhealthy behaviors, all of which increase the risk of morbidity and mortality.	The results emphasize the importance for social and health care policymakers to develop intervention programs to decrease social isolation among older adults by regaining or maintaining social activities and connectedness.
13	Kobayashi, L.C. and Steptoe, A. (2018) England [41]	Social isolation, loneliness, and health behaviors at older ages: Longitudinal cohort study	To examine the among between baseline social isolation, baseline loneliness, and engagement in health behaviors over 10 years among older adults.	Population-based longitudinal cohort study. Social isolation was measure by a five-item index. Loneliness was measured using a three-item short form of the Revised University of California Los Angels Loneliness Scale, while health behaviors were categorized in a binary fashion regarding fruit and vegetable intake, consuming alcoholic drinks and smoking status. The body mass index was also measured.	The ELSA is a population-based longitudinal cohort study of adults aged ≥50 years in England. The cohort began in 2002/2003 based on a random stratified sample of households in England that participated in the Health Survey for England (n = 12,100, response rate = 66%).	Thirteen percent of the participants were socially isolated. These participants were less likely than non-isolated participants to consistently report weekly moderate-to-vigorous physical activity or five daily fruit and vegetable servings. They were less likely to be consistently overweight or obese and more likely to smoke at any time point. Daily alcohol consumption was not associated with social isolation. Loneliness was not associated with health behaviors or the body mass index in adjusted models. Among smokers, loneliness was negatively associated with successful smoking cessation over the follow-up.	Among the sample of older English adults, social isolation may lead to non-engagement in healthy behaviors. Future research should examine whether different modes of social connections influence over health behaviors over time. The results support that social isolation affects health outcomes, such as mortality, through behavioral pathways and warrant the development of psychosocial interventions to improve social isolation and loneliness among older adults.
14	Koyama, S., et al., (2021) England and Japan [42]	Examining the associations between oral health and social isolation: A cross-national comparative study between Japan and England	To examine differences in the association between oral health status and social isolation among older people by comparing Japan and England.	Cross-sectional study from two prospective studies. Social Isolation was measured by the Social Isolation Score with indicators of marital status, presence of children or other immediate family members who provide support, monthly contact with friends, and participation in religious groups, organizations, or committees. Oral health status was measured by the number of remaining teeth or dentures.	Data of adults aged 65 and older were from two ongoing prospective cohort studies including the Japan Gerontological Evaluation Study (JAGES, N = 120,195) and the English Longitudinal Study of Aging (ELSA, N = 3958).	More Japanese participants were socially isolated (1.4% vs. 5.8%) than English participants, but fewer were edentulous (13.1% vs. 7.7%). In both countries, poorer oral health further increased the odds of being socially isolated. Pooled analysis of the ordered logit model with an interaction term showed that the association between the number of remaining teeth and social isolation was stronger in edentulous participants and those in England. In both countries, oral health was associated with social isolation; this association could be stronger in England than in Japan.	Improving oral health could reduce the risk of social isolation among older people. Future studies, such as intervention studies, are needed to validate the positive role of oral health to prevent social isolation in older adults.
15	LeBlanc, R. G., et al., (2022) USA [43]	Social relationship influence on self-care and health among older people living with long term conditions: A mixed-methods study	To determine how social networks influence therapeutic self-care behaviors and health among community-dwelling older people living with multiple long-term conditions.	Cross-sectional explanatory sequential mixed methods design. The quantitative arm consisted of telephone surveys including demographic questions, information regarding chronic conditions and social network functions, measured by the Medical and Outcomes Study: Social Support Survey, and feature numbers in social networks from on the outcomes of self-care, as measured by the Therapeutic Self-care Measure, and health, measured by the Optum SF-12 Health Survey. A nested group (n = 12) from the larger study participated in the qualitative arm of the study involving open-ended interviews.	Community dwelling individuals living in the Northeast U.S. (n = 89) aged 65 years and older, living with two or more chronic conditions, who spoke and understood English, were invited to participate in a telephone survey.	Based on the quantitative results, positive social relationships influenced mental health but not physical health. There was a significant association between social network ties and self-care activities, but no influence on therapeutic self-care medication, symptom, or general health management. Social support influenced therapeutic self-care and mental health. The qualitative results indicated that social networks comprised close friends. Declining physical function influenced the size of social networks, favoring small networks of close relationships. Learning to ask for help was also a theme and was described as a process of learning. Telephone communication was important in providing psycho-social and tangible support.	There are implications regarding the importance of social dimensions in nursing care and community health to consider the person within the context of their social environment. In addition to the telephone as a tool to promote social connections, innovative interventions are needed to promote effect and supportive self-care on both the individual and community level of social networks for individuals with chronic illness.
16	Pohl, J.S., et al., (2022) USA [14]	Social isolation and health among family caregivers of older adults: Less community participation may indicate poor self-reported health	To examine the prevalence of social isolation in a national sample of caregivers, evaluate associations between caregiver social isolation and caregiver health, and explore associations of individual social isolation domain indicators with caregiver health.	This cross-sectional study was a secondary data analysis based on the 2015 National Survey of Caregiving (NSOC) as a supplement to the NHATS study. Self-reported health was measured with a single item. Social isolation was measure based on Berkman and Syme’s Social Network Index.	Among 3501 eligible caregivers with complete data sets for the outcomes, 2186 caregivers, 65 years of age and older, were included.	Nearly 25% of the participants were more socially isolated. Younger caregivers were more isolated compared with those who were not isolated. Self-reported general health was as follows: 4.93% poor; 15.67% fair; 25.62% good; 34.81% very good; and 18.97% excellent. Less social isolation was associated with higher odds of better self-reported health. Those with higher social isolation were more likely to be Black, non-Hispanic, or Hispanic, had less than a college education, and were family members of the care receiver. Of the individual social isolation indicators, only a lack of community participation was associated with higher odds of worse self-reported health. Social isolation and, particularly, community participation were associated with caregiver health status.	It may be necessary for healthcare providers to consider these factors in caregiver health assessments. Future research is recommended to understand the consequences of various social isolation indicators in diverse samples including younger caregivers. Understanding the extent to which caregivers do not participate in their communities is essential. Virtual connections may substitute for face-to- face interaction in protecting against adverse social isolation health outcomes. Further research is needed to focus on the experience of social isolation, particularly for the millennial caregivers who experience high social isolation than older caregivers because of disconnection from friends and who are less satisfied with the quality of their social relationships.
17	Pohl, J., et al., (2017) USA [44]	Measuring Social Isolation in the National Health and Aging Trends Study	To describe the development of a social isolation measure based on Berkman and Syme’s Social Network Index domains with data from the National Health and Aging Trends Study.	Secondary analysis of cross-sectional data from the NHATS using a descriptive correlational design. The Berkman and Syme’s Social Network Index measured social isolation, while the Patient Health Questionnaire-2 measured depression, and an ordinal well-being measure was developed.	Random sample from the Centers for Medicare & Medicaid Services Medicare enrollment database. The sample included 7609 individuals 65 years and older.	The four domains of social isolation included the following: marriage/partner, family/friends, church participation, and club participation. More than half (57%) were married or living with a significant other, and 78% reported talking to family, 18% speaking to friends. Fifty-seven attended church services, and forty percent reported participating in group or club activities. Socially isolated participants were more likely to be older, White non-Hispanic, less educated, and live alone. Participants who were isolated had higher mean depression risk scores and lower mean well-being scores.	The study contributes to science by constructing a measure of social isolation that measures network and integration in a direction that truly reflects isolation. Multiple indicator measures capture important integrating aspects of social isolation rather than using a single item measure.
18	Robins, L.M., et al., (2018) Australia [29]	Social isolation, physical capacity and physical activity in older community-dwelling adults post-hospitalization	To determine whether a relationship exists among physical capacity, physical activity (recreational and/or household-based), and social isolation of older adults recently discharged from the hospital to the community after more than two weeks of hospitalization.	This longitudinal study followed participants for a six-month period post-hospitalization at baseline and 3- and 6-month follow-ups. Measures were the Friendship Scale, Lubben Social Network Scale, and the Australian Survey of Disability, measuring physical capacity, in addition to the Aging and Carers Household, measuring physical capacity. The Phone FITT measured physical activity.	Three hundred and eleven participants were recruited from five hospitals in Victoria, Australia. The majority (58%) were female ranging in age from 65 to 97 years.	Over six months, improvements in physical capacity were related to reduced social isolation. Increased recreational and household-based physical activity were related to contact with more relatives. Between baseline and 3 months, reduced social isolation was related to decreased depression and increases in total physical activity. From Baseline to 3 months and forward to 6 months, increases in the number of friends seen was associated with improved physical capacity and physical activity.	The finding reflects the importance of physical capacity improvements for altering the perception of social isolation among older adults. The results support the need for investigation into physical activity interventions to address social isolation.
19	Salma, J. and Salami, B. (2020) Canada [45]	Growing old is not for the weak of heart: Social isolation and loneliness in Muslim immigrant older adults in Canada	To understand the experiences of healthy aging in Muslim communities in an urban center in Alberta.	A community-based participatory research project was conducted in Alberta, Canada, in 2017–2018. The research questions were as follows: (a) tell me about the social connections in your everyday life; (b) describe your daily social activities and daily recreation; and (c) describe the challenges you experience in staying connected to family, friends, and the community.	In total, 67 older adults (mostly women) and stakeholders from South Asian, Arab, and African Muslim communities participated in one of twenty-three individual interviews or seven focus group discussions over a 1-year period.	The themes identified include the intersection of exclusion based on agism, racism and sexism, and strategies for inclusion at local, national, and transnational levels as counter-approaches to social isolation. Social isolation was a common experience in Muslin older adults whether they arrived in Canada recently or decades ago. There was a lack of ability to connect with peers the same age, though they connected with children and grandchildren. Being older adults and minorities excluded participants from particular social spaces, both public and private. Experiences of racism and discrimination limited welcoming spaces. Older Muslim women experienced gender-based discrimination both within their communities and from mainstream society. Strategies for inclusion included supporting long-term integration in Canada for immigrant newcomers. Also recommended were creating welcoming and safe spaces where older adults could interact with people from other cultures and religions, as well as those who share the same language and culture. Participants sought to develop programs in their communities that combine socialization, physical activity, and prayers for older Muslin adults who could travel may visit family in their country of origin. For those who could not travel, the recommendation was to increase connections through multimedia such as phone calls, increase connections through phone calls or use of social media.	The study findings point to the intersecting influences of exclusionary practices on social isolation and loneliness in immigrant older adults. The results highlight areas for intervention to strengthen personal and community level agency. Senior programs would include opportunities to socialize with peers that share language and cultural and religious heritage in addition to opportunities for socialization within mainstream society. Volunteer and employment opportunities would be of value to increase socialization.
20	Schrempft, S., et al., (2019) London [25]	Associations between social isolation, loneliness, and objective physical activity in older men and women	To test the hypothesis that social isolation and loneliness are associated with less objective physical activity and more sedentary behavior in older adults. To measure physical activity, wrist-mounted accelerometers were worn for 7 days.	A cross sectional study. Associations between social isolation or loneliness and objective activity were analyzed using linear regressions. Social isolation and loneliness were assessed with standard questionnaires, and poor health, mobility limitations, and depressive symptoms were included as covariates.	The sample consisted of 267 community-based men (n = 136) and women (n = 131) aged 50–81 years (mean 66.01), who took part in the English Longitudinal Study of Aging (ELSA; Wave 6, 2012–13).	Total 24-hour activity counts were lower in isolated compared with non-isolated respondents independent of gender, age, socioeconomic status, marital status, smoking, alcohol consumption, self-rated health, limiting longstanding illness, mobility limitations, depressive symptoms, and loneliness. Time spent in sedentary behavior over the day and evening was greater in isolated participants, while light and moderate/vigorous physical activity were less frequent. Physical activity was greater on weekdays than weekend days, but associations with social isolation were similar.	These findings suggest that greater social isolation in older men and women is related to reduced everyday objective physical activity and greater sedentary time. Differences in physical activity may contribute to the increased risk of ill-health and poor well-being associated with isolation.
21	Silberman-Beltramella, M., et al., (2022) Spain [46]	Social relations and health in older people in Spain using SHARE survey data	To describe social relations in individuals over age 50 in Spain and analyze their association with physical/emotional, functional, and cognitive/sensory health variables.	Cross-sectional study based on a sample from wave 6, collected in 2015, in the Survey of Health, Aging, and Retirement in Europe (SHARE), which was a longitudinal, multidisciplinary study on the health, economic status, and social and family networks of more than 140,000 individuals from 27 countries in Europe and Israel. Socio-demographic variables were collected. Physical and emotional health included disease diagnoses and number of medications taken. SHARE uses the U.S. version of the Short-Form Health Survey (SF-36) and the EURO-D depression Scale. Basic activities of daily living were collected. Cognitive and sensory variables were also included. Social relations were measured by the R-UCLA Short Loneliness Scare.	Using multistage sampling, 5583 individuals were selected from a representative sample of the Spanish population aged 50 and over, who were contacted for an interview with both the selected individuals and their partners if they lived in the same household.	The average age of participants was 70 years of age, and 54% were female. The degree of satisfaction with social relationships was high at 68%. The majority (67%) were not lonely, and 95% had family in their social network, while 78% said that they did not have a friend. Physical and emotional health was significantly higher in people who were in social relationships. Functional ability was related to the perception of loneliness. Cognitive and sensory ability were higher in those who were not lonely. The study indicated that people who feel more lonely are those with poorer physical and emotional health.	The study suggests that social relation characteristics, measured by network size, as well as satisfaction and intensity, measured as the perception of loneliness, should be acted upon by multidisciplinary involvement to promote the health of older adults. Facilitating social support for the loneliest older adults who receive no help from their social networks, and encouraging healthy activities that enable them to strike up and strengthen friendships are possible solutions to the problem of loneliness in older adults is required. Action should also be taken to reinforce both home care strategies and the role of the liaison nurses, not only in managing available resources but also in encouraging family involvement and communication to obtain cost-effective and quality results.
22	Smith, K.J. and Victor, C. (2019) UK [47]	Typologies of loneliness, living alone and social isolation, and their associations with physical and mental health	To explore typologies based on shared experiences of loneliness, social isolation, and living alone using Latent Class Analysis and determine how these groups may differ in terms of their physical and mental health.	Longitudinal study based on data collected from the English Longitudinal Study of Aging (ELSA), which is a Health Survey for England in either 1998, 1999, or 2001. The scales used were the UCLA Loneliness Scale, and social isolation was based on household composition, participation in social activities, and communication with family, relatives and friends. Socio-demographic characteristics were assessed. Function was assessed by calculating activities of daily living, a list of chronic conditions was assessed, and self-rated health was measured as a single item. Depressive symptoms were measured by the Center for Epidemiological Studies Depression Scale.	Participants were from Wave 7, the most recently published wave of ELSA. A total of 8249 people took part in the wave. Following exclusion criteria, the sample consisted of 7032 participants (mean age = 67.3; 55% female).	A six-cluster topography was identified which included (1) no loneliness or isolation; (2) moderate loneliness; (3) living alone; (4) moderate isolation; (5) moderate isolation, living alone; and (6) high loneliness moderate isolation with high likelihood of living alone. Groups experiencing loneliness and/or social isolation reported poorer physical and emotional health, even after controlling for socio-demographic confounders. Poor health was also a risk factor in developing loneliness. The results indicated that living alone was conceptually separate from loneliness and isolation and had limited utility as a measure of these complex concepts.	Using Latent Class Analysis (LCA) uncovered different groups based on shared experiences of loneliness, social isolation, and living alone, showing the different experiences of older adults. The work indicates that the lived experiences of loneliness, social isolation, and living alone in older adults is complex and that taking the number of issues and severity of issues into account will be important for researchers and clinicians working with groups of older adults who may experience these issues.
23	Zhang, D., et al., (2022) China [48]	What could interfere with a good night’s sleep? The risks of social isolation, poor physical and psychological health among older adults in China	To provide one of the first population-based longitudinal studies investigating the association between social isolation and sleep difficulty among adults age 60 years and older. Three major research questions were investigated as follows: (1) does the risk of sleep difficulty in later life vary by older adults’ social isolation status experienced in daily life? (2) to what extent are the associations between social isolation and sleep difficulty mediated by the psychological and physical well-being of an older adult? and (3) do existing psychological and physical problems exacerbate the association between social isolation and sleep difficulty?	Population-based longitudinal studies with five cognition-related questions drawn from the Mini-Mental State Examination (MMSE). Sleep difficulty was measured as a single item. Social isolation was measured using the Lubben Social Network Scale. Psychological being was measured by the depressive symptoms on the CED-D scale. Physical well-being indicators were self-rated pain and chronic diseases.	The sample included 8456 community-dwelling participants from the China Longitudinal Aging Social Survey (CLASS, 2014, 2016, and 2018). The participants were adults aged 60 years and older from 28 provinces, autonomous regions, and municipalities in mainland China.	There was clear evidence that social isolation (family/friendship ties) is an independent risk factor for sleep difficulty. Social isolation was positively associated with higher depressive symptoms, greater pain, and more chronic diseases The risk of sleep difficulty was especially pronounced for older adults who were both socially isolated and suffered from multiple chronic diseases	Future research with more detailed information on multiple social relationships and sleep outcomes should continue to explore how older adult isolation from different types of relationships may have adverse impacts on sleep health. These findings have important implications for both scientific understandings and effective prevention of sleep problems among older adults in China. Researchers need to step away from a narrow family network when examining social relationships and their associations with sleep health. Policy interventions that integrate social connections from the broader social networks, such as friends and neighbors, would be critical. Policymakers should commit resources to provide older Chinese adults with more opportunities to participate in various social activities in the community and activate social interactions among those with limited social networks.
24	Freak-Poli, R., et al., (2022) UK [49]	Social isolation, social support and loneliness as independent concepts, and their relationship with health-related quality of life among older women	To assess whether social isolation, social support, and loneliness are independently associated with health-related quality of life of adult Australian women aged 70–75.	Secondary retrospective analysis of women aged 70–75 years from the Australian Longitudinal Study on Women’s Health (ALSWH). Social isolation, social support (Duke Social Support Index), and loneliness (single item) were investigated for their association with Health-Related Quality of Life (HRQOL) and physical [PCS] and mental [MCS] components of the SF-36 questionnaire. Analyses were adjusted for sociodemographic variables and the number of medical conditions.	The sample included 10,517 women aged 70–75 years from the Australian Longitudinal Study on Women’s Health (ALSWH).	Among the women, 61% were socially isolated, 9% had low social support, and 14% were lonely. Those with social isolation and low social support reported being lonely. There were strong inverse associations among social isolation, social support and loneliness, and mental and physical health. Each construct was independently associated with HRQoL, with loneliness having the strongest inverse association.	Among older women, social isolation, low social support and loneliness are distinct, partially overlapping yet interconnected concepts that coexist and are each adversely associated with HRQoL. Interventions targeting loneliness may have the greatest benefit for health outcomes. The study highlights the need for clinicians, health services and governments to give priority to assess social isolation, social support, and loneliness and develop large scale strategies to minimize their adverse outcomes in older adults.
